# EQ-5D-5L Slovenian population norms

**DOI:** 10.1186/s12955-020-01584-w

**Published:** 2020-10-07

**Authors:** Valentina Prevolnik Rupel, Marko Ogorevc

**Affiliations:** Institute for Economic Research, Kardeljeva ploščad 17, 1000 Ljubljana, Slovenia

**Keywords:** EQ-5D-5L, EQ VAS, Population norms, Reference values, Health status, Patient-reported outcomes

## Abstract

**Background:**

The study aims to present Slovenian EQ-5D-5L population norms for different age and gender subgroups that can be used as reference values in future studies concerning health status. The secondary aim is to compare those norms with population norms from some other countries in Europe and elsewhere.

**Methods:**

The cross-sectional survey was conducted between November 2019 and February 2020 via online panel. 1071 adults aged 18+ were included in the survey. The general population was sampled using quota sampling in terms of age, gender, and NUTS2 region. In the study, the EQ-5D-5L Slovenian online version was used. Descriptive statistics was used to present health status by age groups and genders for the EQ-5D-5L descriptive system, EQ VAS and the EQ-5D-5L index score. The latter was derived from Slovenian EQ-3D-3L tariff, transformed to five levels using the crosswalk methodology.

**Results:**

The mean EQ VAS score in the Slovenian population was 79.9, mean utility index was 0.808. 28.2% of the population did not have problems on any dimension and 3.9% of the population had problems on all dimensions. Persons residing in Western Slovenia had, on average, 0.016 higher utility score, compared to Eastern Slovenia. Effect of gender was not significant. Age was negatively associated with both utility index and EQ VAS score. Education was positively correlated to health status. Problems on dimensions were generally increasing with age, except for anxiety/depression dimension, where youngest group (ages 18–29) reported more anxiety/depression compared to older counterparts. Self-reported anxiety/depression was more pronounced in women.

**Conclusions:**

Similarly to other countries, the health generally deteriorates with age, except for the anxiety/depression dimension where the share of respondents reporting no problems was lowest in the youngest age group. The open question for the future remains, whether population norms from this online sample differ significantly from the actual EQ-5D-5L health status data of the Slovenian general population.

## Background

While every one of us can casually and without insight into medical knowledge estimate own health at the moment, measuring health and being healthy is relative and depends on many factors such as age, gender, education, health state a day before, the experience of disease in self and others. Still, for the last three decades, many ways to measure health were invented, underlying reason being the need for data to guide efforts toward reducing the consequences of disease and enhancing the benefits of good health.

Many valid and reliable instruments exist with the purpose of measuring health. They can be grouped into generic and disease-specific instruments [1]. Generic instruments measure general health status via various dimensions such as physical symptoms, function and the emotional dimensions of health. As these dimensions are relevant to all health states, those can be compared across different diseases this being main advantage of the generic questionnaires [2]. Some of the most prominent generic questionnaires are the Health Utility Index (HUI) [3], the Short Form 6D [4], 15D instrument [5], Assessment of Quality of Life (AQOL) [6], the Sickness Impact Profile (SIP) [7] and the EQ-5D-5L [8].

Disease-specific instruments, on the other hand, measure quality of life for a specific disease, for which they are designed. They are more sensitive to assess changes within patients. However, in line with their purpose, the results are disease-specific and cannot be compared between populations with different diseases [9].

The EQ-5D is a generic preference-based measure developed by the EuroQol Group [10]. It has been suggested to be the one of the most commonly used preference-based measures in the world. Despite this, the validity of the first version of EQ-5D, namely 3-level version (EQ-5D-3L), was hampered by a ceiling effect [11]. To avoid that limitation, EQ-5D-5L was developed in 2009. By now it has been tested in different samples, showing strong psychometric properties [12, 13]. The new version kept its original five dimensions but expanded the response options from 3 to 5 levels. Furthermore, the response options in EQ-5D-5L changed from previously being lower (no problems, some problems, a lot of problems) to a higher level descriptive system (no problems, slight problems, moderate problems, severe problems and unable/extreme problems).

Population norms enable the comparison of the citizens’ health status both within a society among different subpopulations as well as between countries. When estimating disease burden, the health status of specific patient groups is compared to the population norms of the general public with similar socio-demographic characteristics, predominantly age and gender [14]. In the absence of a country specific EQ-5D population norm, norms from other countries can be used on the condition of taking into account intercountry difference. The application of an arbitrary dataset without adjustments might lead to biases, and finally, to poor health policy decisions. The scarcity of EQ-5D population norms in the Central and East European (CEE) region is a concern from both public health and HTA perspective [15].

The study aims to present Slovenian EQ-5D-5L population norms for different age and gender subgroups that can be used as reference values in future studies concerning health-related quality of life. The secondary aim is to compare those norms with population norms from some other countries in the CEE region and elsewhere.

## Methods

The following section presents data collection, sample size, and participant recruitment for the EQ-5D-5L questionnaire, together with a short description of methods for data analysis.

### Data collection

The data on the preferences of adults towards health states were collected in the IMPACT HTA study, with a goal to explore whether values for health states for children (11–17 years of age) estimated by the general population (age 18+) differ from values obtained from adolescents themselves for the same health states. In this context, standard methods to elicit preferences, namely the DCE (discrete choice experiment) valuations for EQ-5D-Y-3L health states in combination with TTO (time trade-off) were applied to collect the preferences from the respondents with the aim to develop EQ-5D-Y-3L value set.

Besides the DCE task, each respondent reported his/her self-reported health using EQ-5D-Y-3L and EQ-5D-5L profiles alongside the EQ VAS rating. The questionnaire also included some demographic and social-economic questions. While EQ-5D-Y-3L questionnaire was positioned in front of DCE task, EQ-5D-5L profile alongside EQ VAS rating was positioned after the DCE task and in front of social and demographic questions. The study ran simultaneously in Germany, Spain, and Slovenia.

The survey was conducted via a statistical survey web application LimeSurvey. Participants from the online panel were recruited and contacted via email by Slovenian market research company Valicon, the owner of the online panel. The email contained an access link to the online survey. Before the start of the study, the participants needed to give informed consent within the software. Data collection started in November 2019 and finished in the end of February 2020.

### The EQ-5D-5L Questionnaire

In the study, the EQ-5D-5L Slovenian online self-complete Laptop/Desktop version was used [10]. The EQ-5D-5L descriptive system consists of the five dimensions: mobility (MO), self-care (SC), usual activities (UA), pain/discomfort (PD), and anxiety/depression (AD). In each dimension, respondents are asked to describe their current health with one of the following five levels of severity: no problems, slight problems, moderate problems, severe problems, and unable/extreme problems [16]. The descriptive system defines 3,125 (5^5^) distinct health-states. Responses from the five above mentioned dimensions can be combined in a single health state in a form of a 5-digit number (the best state “11111” meaning no problems at any of dimension to the worst state “55555” meaning unable/extreme problems at all five dimensions). The second part of the instrument is a 20-cm visual analog scale (EQ VAS) ranging from 0 (worst health you can imagine) to 100 (best health you can imagine), which serves for estimation of individual general health status.

### Data analysis

The descriptive statistics are presented for the whole sample as well as for the predefined age groups (18–29, 30–39, 40–49, 50–59, 60–69, 70+) and gender. EQ-5D-5L utility index was derived from Slovenian EQ-3D-3L tariff [17], transformed to 5L using a probability matrix—the “crosswalk” methodology as described and applied in Golicki et al. [18].

Data analysis was done using R [19].

### Ethical approval

The research study was approved by the National Medical Ethics Committee of Slovenia, number 0120-154/2018/08, on 24 May 2018. The amendment to the study was approved by the National Medical Ethics Committee of Slovenia, number 0120-154/2018/15, on October 16, 2019.

## Results

**Fig. 1:**
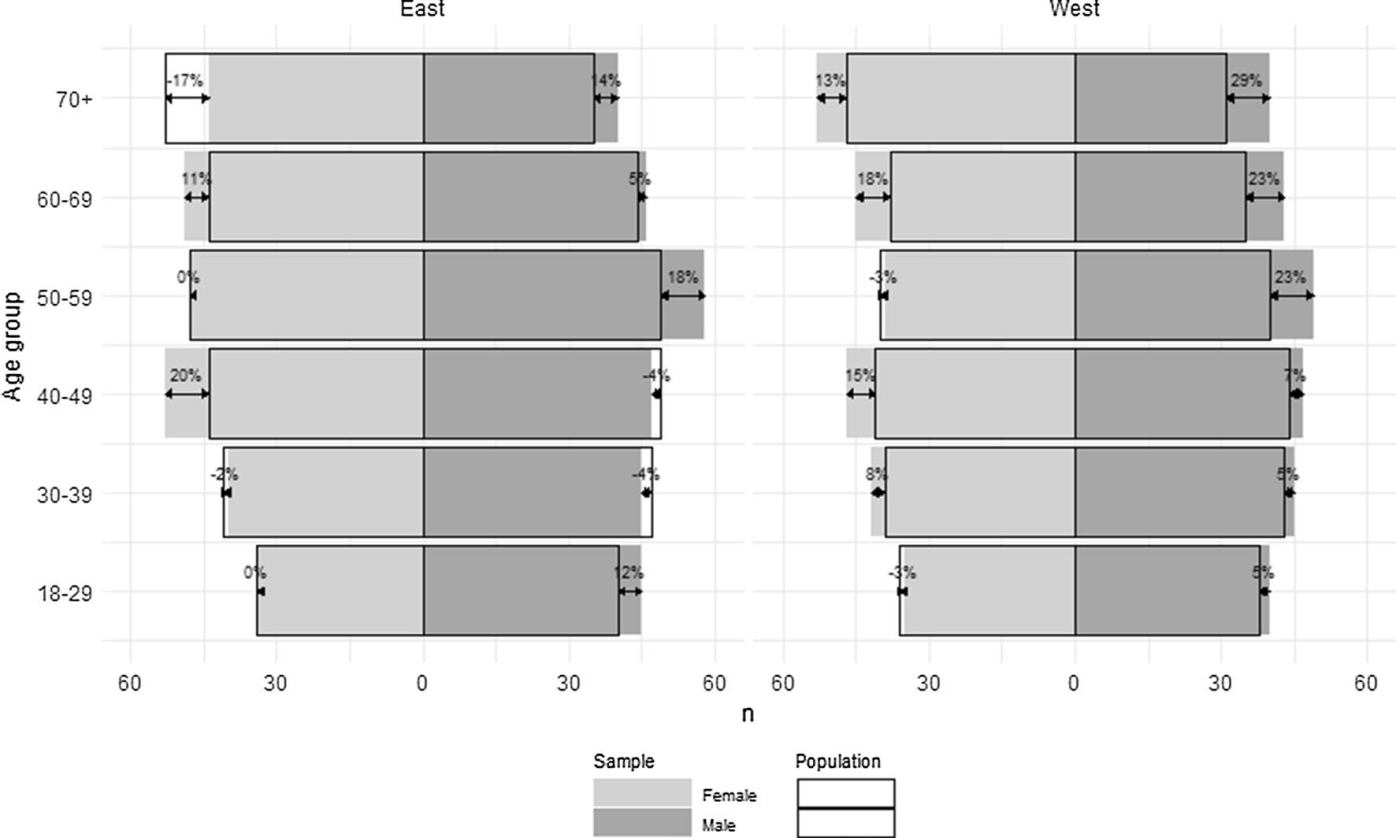
Sample quotas. *Source*: Author’s own calculations

Invitation to participate in the study was sent to 2264 adults, out of whom 763 did not respond and 430 did not complete the survey. Overall, a total of 1071 adults aged 18+ completed the survey, 527 male and 544 female. The general population was sampled using quota sampling in terms of age, gender, and NUTS2 region, as shown in Fig. 1.

Boxes represent quotas for sub-populations, while grey bars represent the actual sample. The quotas were not reached in group “Females over 70 years in Eastern Slovenia” (− 17%). Some other quotas were over-represented, such as females aged 40–49 in Eastern and Western Slovenia, males aged 50–59 in Eastern and Western Slovenia and all gender and regional groups aged 60–69. Participants in the online survey were more educated compared to general population (Primary—10%, Secondary 55.8%, Tertiary—33.4%). Their mean EQ VAS score was 79.9, mean utility 0.808. Sample statistics are presented in Table 1.

**Table 1:** Descriptive statistics. *Source*: Authors’ own calculations

Variable	Values	Frequency/stats	Graph
Gender	Female	527 (49.2%)	
	Male	544 (50.8%)	
Education	Primary	27 (2.5%)	
	Secondary	356 (33.3%)	
	Tertiary	685 (64.1%)	
Age group	18–29	153 (14.3%)	
	30–39	172 (16.1%)	
	40–49	194 (18.1%)	
	50–59	193 (18.0%)	
	60–69	182 (17.0%)	
	70 +	177 (16.5%)	
EQ VAS		Mean (sd): 79.9 (15.8) min < med < max: 0.0 < 83.0 < 100.0 IQR (CV): 19 (0.2)	
Utility *index*		Mean (sd): 0.808 (0.160) min < med < max: 0.243 < 0.836 < 1.000 IQR IQR (CV): 0.331 (0.198)	

Overall, 26.9% of population has at least slight problems with walking about (mobility), 7.4% has at least slight problems with washing and dressing themselves (self-care), 21.9% has at least slight problems doing their usual activities, 58.1% have at least some pain, and 38.9% are at least slightly anxious or depressed. A full description of problems by health dimensions is presented in Table 2 (breakdown by gender and age groups in the appendices).

**Table 2:** Percentages of population with various levels of problems across EQ-5D dimensions. *Source:* Authors’ own calculations

Variable	Stats/values	Frequency	Graph
Mobility (MO)	1. No problems	783 (73.1%)	
	2. Slight problems	209 (19.5%)	
	3. Moderate	63 (5.9%)	
	4. Severe problems	16 (1.5%)	
	5. Unable	0 (0%)	
Self-care (SC)	1. No problems	992 (92.6%)	
	2. Slight problems	60 (5.6%)	
	3. Moderate	16 (1.5%)	
	4. Severe problems	3 (0.3%)	
	5. Unable	0 (0%)	
Usual Activities (UA)	1. No problems	836 (78.1%)	
	2. Slight problems	177 (16.5%)	
	3. Moderate problems	44 (4.1%)	
	4. Severe problems	13 (1.2%)	
	5. Unable	1 (0.1%)	
Pain Discomfort (PD)	1. No pain	449 (41.9%)	
	2. Slight pain	474 (44.3%)	
	3. Moderate pain	124 (11.6%)	
	4. Severe pain	24 (2.2%)	
	5. Extreme pain	0 (0%)	
Anxiety Depression (AD)	1. Not anxious	663 (61.9%)	
	2. Slightly anxious	310 (28.9%)	
	3. Moderately anxious	75 (7.0%)	
	4. Severely anxious	15 (1.4%)	
	5. Extremely anxious	8 (0.8%)	

28.2% of the population does not have problems on any dimension and 3.9% of the population had problems on all dimensions. Differences in EQ-5D-5L index, EQ VAS scores and severity of anxiety) with respect to demographic characteristics were explained with general multivariate regression models. Severity of anxiety is treated as numerical variable ranging from 2 to 5 (slight anxiety/depression to extreme anxiety/depression). Results are presented in Table 3.

**Table 3:** HRQoL differences by demographic characteristics. *Source:* Author’s own calculations

	Dependent variable		
	Utility index	EQ VAS score	Severity of Anxiety^a^
Slovenia—West	0.016*	0.58	− 0.018
	(0.009)	(0.96)	(0.063)
Gender—Male	0.005	− 1.06	0.137**
	(0.010)	(0.96)	(0.064)
Age 30–39	0.003	0.77	− 0.287***
	(0.017)	(1.74)	(0.105)
Age 40–49	0.007	0.54	− 0.393***
	(0.017)	(1.69)	(0.104)
Age 50–59	− 0.040**	− 0.88	− 0.376***
	(0.017)	(1.69)	(0.104)
Age 60–69	− 0.072***	− 3.96**	− 0.200*
	(0.017)	(1.72)	(0.108)
Age 70 +	− 0.086***	− 7.13***	− 0.199*
	(0.017)	(1.73)	(0.107)
Secondary education	0.073**	6.23**	0.084
	(0.031)	(3.13)	(0.209)
Tertiary education	0.102***	6.72**	0.019
	(0.030)	(3.07)	(0.206)
Constant	0.740***	75.58***	2.459***
	(0.034)	(3.38)	(0.221)
Observations	1,068^b^	1,068^b^	406
R^2^	0.072	0.038	0.062
Adjusted R^2^	0.064	0.030	0.040
Residual St.e. (df = 1058)	0.155	15.573	0.627 (df = 396)
F Statistic (df = 9; 1058)	9.114***	4.630***	2.885*** (df = 9; 396)

*p < 0.1; **p < 0.05; ***p < 0.01

^a^At least slight depression, standard errors in brackets, ^b^3 observations omitted due to missing education

Persons residing in Western Slovenia had, on average, 0.016 higher utility score, compared to Eastern Slovenia, *ceteris paribus*, whereas location was not correlated with VAS score. Effect of gender was not significant. Age was negatively associated with both utility index and VAS score. Both indicators decreased with age, from 81.8 (age group 18–29) to 74.7 (age group 70+) or 0.84 to 0.756 measured by utility index. Persons aged over 50 reported, on average, a lower score. Age group 70 + had 0.086 lower utility index, and 7.2 lower rating on VAS, compared to the youngest group (18–29) (Table 4). Moreover, education was positively correlated to both EQ-5D-5L index and EQ VAS. Persons with higher than primary education had higher quality of life measured both with utility index and VAS.

**Fig. 2:**
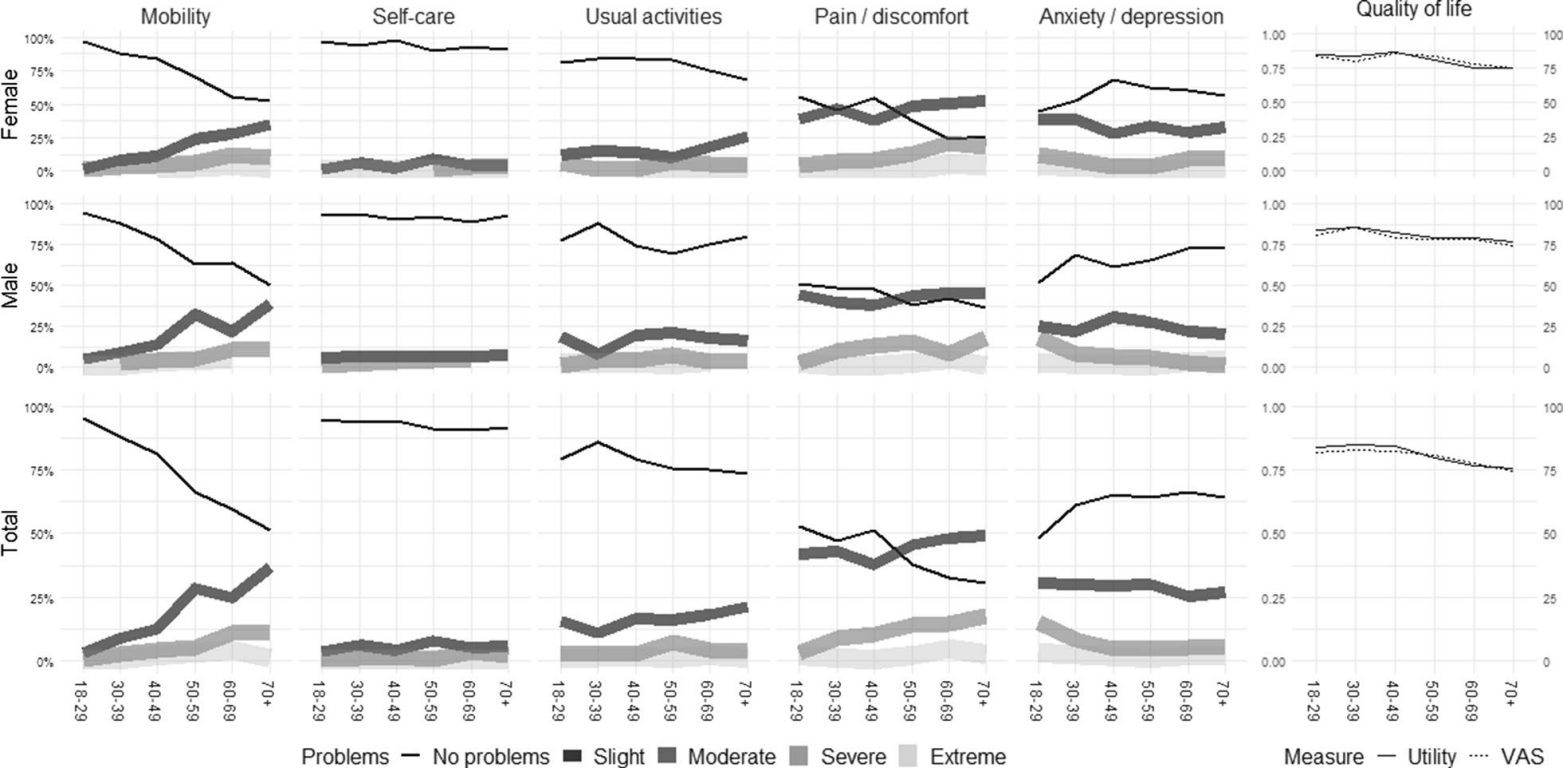
Quality of life by age and gender. *Source*: Authors’ own calculations

Problems on dimensions are generally increasing with age, except for AD dimension (see Fig. 2), where the trend is reversed. The youngest part of the adult population (ages 18–29) reported more AD compared to older counterparts. Surprisingly, younger people report more “moderate AD” compared to other age groups (see Table 2 and Fig. 2). Self-reported AD is more pronounced in women. 33% of women and 25% of men report slight AD, while 9% of women and 18% of men aged 18 to 29 report moderate AD. Older generations report moderate AD in 6 to 8% (see Figs. 4, 5, 6). Findings were additionally confirmed with a regression model, where dependent variable was severity level of AD (see Table 2).

The most rapid and significant decrease of “no problems” is visible in the MO dimension, where with age people move from “no problems” to mainly “slight problems” in walking, and “moderate problems” to some extent. Slight PD is reported in 44% of the general population and is increasing with age. The younger population reported slight PD in 42%, and moderate PD in 3%, while people aged over 70 reports slight PD in 49% and moderate PD in 18% of the cases. There is a peak in PD dimension in the age group 40–49 with 51% of population reporting no problems in comparison to 47% in age group 30–39. Looking at the increase in men and women separately, it is fully a consequence of higher percentage of women aged 40–49 reporting no problems with PD (55% in comparison to 45% reporting no problems in age group 30–39). Similarly, a peak in respondents reporting no problems can be observed in UA dimension in age group 30–39 (Figs. 4, 5, 6). This increase is largely driven by men: 88% of men aged 30–39 report no problems in comparison to 78% of men aged 20–29. The increase in the same age group on UA dimension is observed is women as well (81% vs 84%).

In order to put reported problems in a broader context, a quick literature review was performed and accessible population norms reports from the following countries were looked at: China (urban areas) [20], Germany [21], Indonesia [22], Italy [23], South Korea [13] Poland [2], Quebec [24], Spain [25], Thailand [26], Trinidad and Tobago [27], Uruguay [28] and Vietnam [29].

**Fig. 3:**
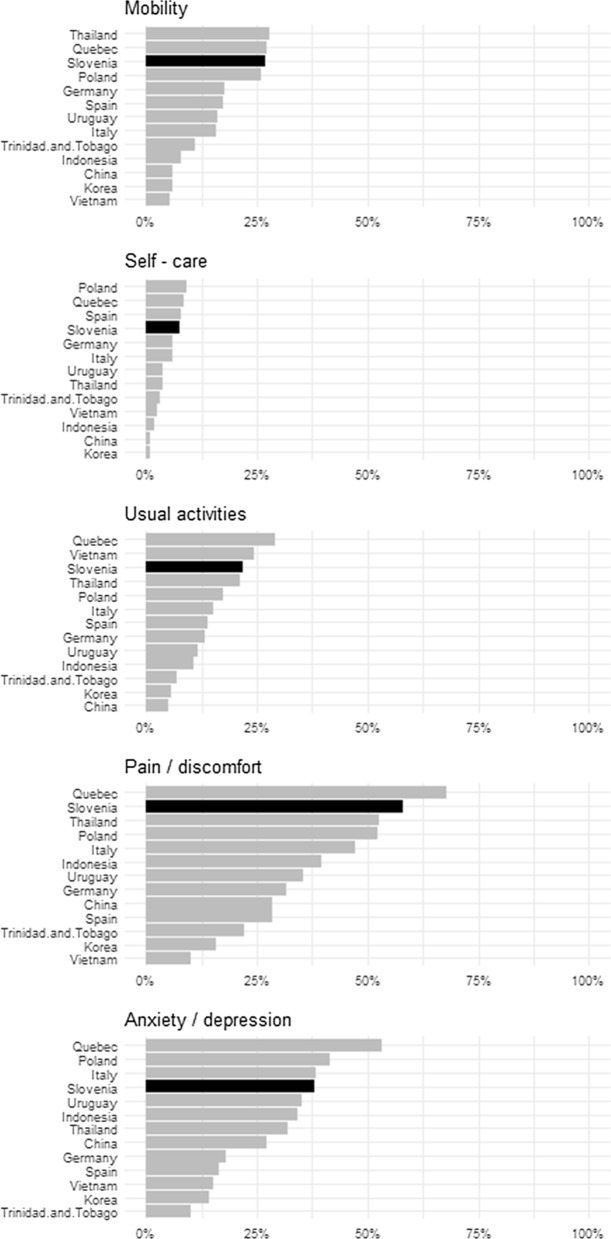
Prevalence of problems by country (at least slight problems). *Source*: Augustovski et al. [28], Bailey et al. [27], Garcia-Gordillo et al. [25], Golicki and Niewada [2], Hinz et al. [21], Kim et al. [13], Nguyen et al. [29], Pattanaphesaj et al. [26], Poder et al. [24], Purba et al. [22], Scalone et al. [23], Yang et al. [20], Authors’ own calculations

**Fig. 4:**
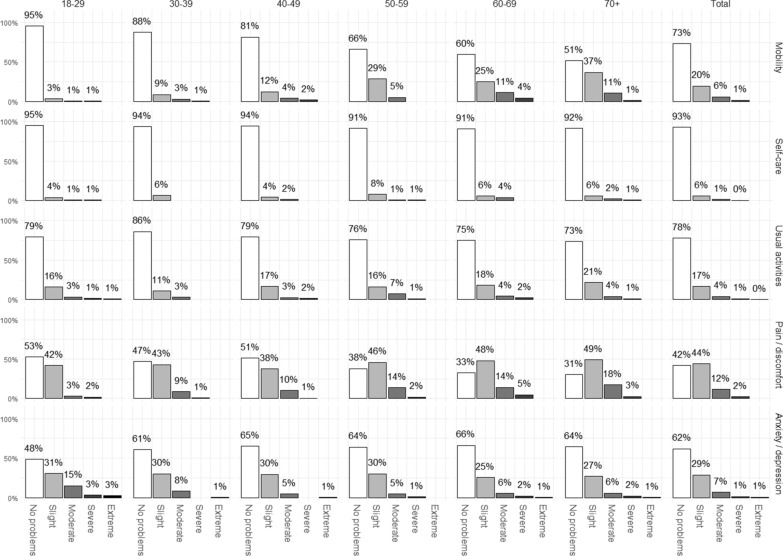
Problems reported by dimension, problem level and age group, general population

**Fig. 5:**
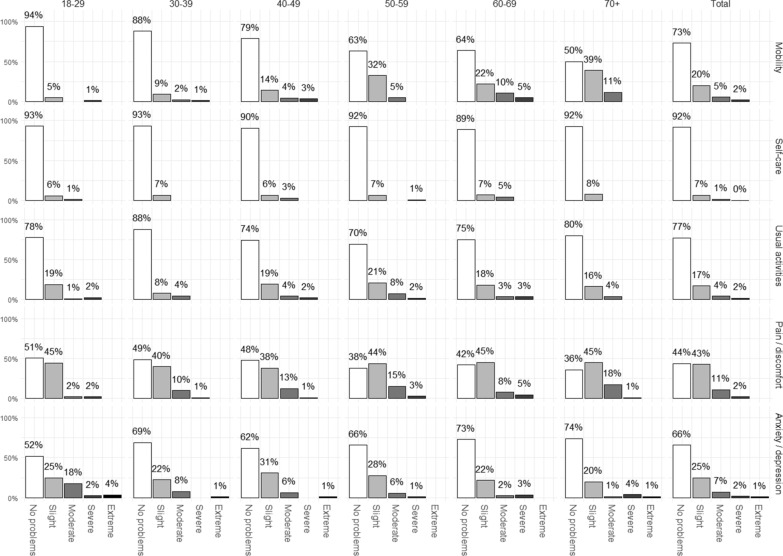
Problems reported by dimension, problem level and age group in men

**Fig. 6:**
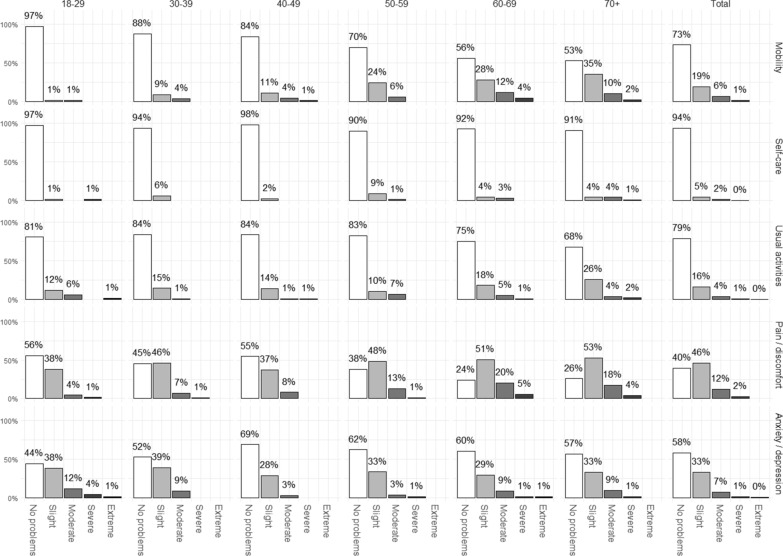
Problems reported by dimension, problem level and age group in women

Slovenian self-reported problems on health dimensions rank relatively high compared to other countries, as Slovenia ranks among top 4 countries on all dimensions. Correlation-wise, Slovenian values are most similar to Thailand, Quebec and Poland. In all selected countries and Slovenia, the prevalence of the problems is the lowest in self-care dimension: no problems are reported in at least 90.0% of population up to 98.92%. The dimension with the highest prevalence of problems is pain/disability in most of the countries, including Slovenia (with the exception of Indonesia where the highest share of reported problems is in AD dimension and Vietnam in UA dimension) (Fig. 3). With regards to AD problems in younger generation, the results across the countries differ a lot. While in some countries the share of youngest reporting problems in AD dimension is low (e.g. Vietnam 13.9%, Spain 6.4%), the shares in some other countries are much higher (e.g. 34.5% for male and 37.2% for female in China, 40.1% in Indonesia and 40.3% for female in Italy), although they still do not reach the level in Slovenia, where 52% of the youngest age group report having problems with AD dimension (56% female and 48% male). In Slovenia the percentage of those reporting problems in AD is generally decreasing with age, up to the age of 40, and is lower in all age groups than in the youngest: similar situation is seen in China, Indonesia and for female in Italy. In comparison to Poland as the only other country in the same geographical area, such trend is not noticed and problems with AD dimension decrease with age [2, 13, 20–29] (Figs. 4, 5, 6).

## Discussion

The Slovenian EQ-5D-5L population norms were estimated on a quota sample of Slovenian adults aged 18+. The sample is highly representative of age, gender as well as NUTS-2 level regions in Slovenia. Size as well as representativeness and reliability of the sample surely are among the strengths of the study. The study took place simultaneously in three countries using a coherent design and same set-up. The questionnaire used is the official EQ-5D-5L version of the EQ-5D questionnaire, approved by EuroQol office.

Among the weaknesses that need to be presented is first of all the fact, that study lacks representativeness in terms of education which was not included as a sampling quota criterion. Higher education of the respondents in comparison to general population, alongside other variables which were not included as a sampling quota criterion, may result in different preferences towards health states. Additionally, the study was conducted online. During the data collection process, youngest and oldest age groups were most difficult to reach and recruit. For the elderly age groups the difficulties could be ascribed to less computer skills in these age groups. As the questionnaire was formatted for use on desktop/laptop, but not for use on mobile phones, this could be one of the reasons for harder recruitment process among the youngest groups of participants. Furthermore, the respondents were asked to participate in a valuation study using DCE, which is a challenging task and could introduce a selection bias in terms of willingness to participate. However, a large majority of the participants who did not complete the study once started were reportedly using mobile phone which made it impossible for them to finish the study. It can be concluded, therefore, that the difficulty of the task itself did not impact the sample to a large extent. Lastly, the valuation exercise in a health state valuation study may influence respondents' thoughts about their own health and alter the results of a self-reported health questionnaire. As EQ-5D-5L profile and EQ VAS task were positioned after the valuation task within the online survey, this might have influence the results. In the study, EQ VAS and EQ-5D-5L utility index values were estimated for the general Slovenian population. To estimate the latter, Slovenian interim value set, based on the crosswalk methodology was used. Directly measured and estimated Slovenian value set would be preferred, however, the study of eliciting preferences of general population towards EQ-5D-5L health states has not been conducted yet.

Similarly to other countries [2, 13, 20–29], the health generally decreases with age. The surprising exception in Slovenian data is the AD dimension where the share of respondents reporting no problems was lowest in the youngest age group and was increasing by age until the age of 40, after which it remained stable. Same phenomenon can be observed in China, Indonesia and for women in Italy. It is difficult to reason why youngest in these countries would report problems with AD in such high shares. However, this is an important feature to consider in other countries in the region without own population norms.

The health state on this same dimension was worse for women. The number of reported limitations by women on AD dimension was confirmed also in Poland and China (alongside PD) [2, 20] in South Korea (alongside with PD and MO) [13] or in Italy (alongside PD, MO and UA) [23]. A 10 percentage point increase in women reporting no problems with PD in age group of 40–49 indicates that women in this age group have less problems on this dimension than expected. A further research could explain the underlying reasons. An increase in respondents reporting no problems in UA dimension in age group 30–39 in comparison to 20–29 could be ascribed to low percentage of population reporting no problems in UA dimension among the youngest. In other countries, the percentages of youngest reporting no problems with UA are higher (96.5% in Poland, 88.54% in Indonesia, 98.4% in Spain, 96.0% and 91.2% for men and women in Italy and 96.4% and 96.2% for men and women in China) compared to Slovenia (79% total, 78% men and 81% women) [2, 13, 20–29] (Figs. 4, 5, 6). Again, the explanation as of why such a high percentage of youngest in Slovenia would report problems with usual activities, is unclear and further research is needed to explore the issue.

There are many ways for the potential use of the results in health care policy. The clinicians can use the population norms reference to compare the information on health state of patients with the health state of general population within same gender and age groups [30]. The data can also be used for the assessment of health of the Slovenian population as well as to conduct cross-country or cross-regional comparisons. Other stakeholders, such as health economists, can use population norms in the adaptation of health economic models to Slovenia.

Future studies in Slovenia include EQ-5D-5L valuation study based on a direct valuations for adults as well as estimation of EQ-5D-Y-3L value set. Further research into AD dimension is needed. Also, it is necessary to ascertain in the future studies whether population norms from this online sample differ significantly from the actual EQ-5D-5L health status data of the Slovenian general population—in such endeavors this sample can serve as a valuable source of reference scores.

## Conclusions

The study presents EQ-5D-5L population norms for Slovenia. The presented norms should encouraged clinicians, economists and policy makers in Slovenia to use this instrument in combination with disease-specific instruments on a wider scale. Further research into more representative population is warranted.

## Data Availability

The datasets generated and/or analyzed during the current study were obtained within IMPACT HTA study. After the conclusion of the project the data will be available in the Zenodo repository.

## Appendix 1

See Fig. 4.

## Appendix 2

See Fig. 5.

## Appendix 3

See Fig. 6.

## Appendix 4

See Table 4.

**Table 4:** Health related quality of life by age group and gender

Gender	Measure	Age group						
		18–29	30–39	40–49	50–59	60–69	70 +	Total
Female	VAS	83	79.6	84.6	83	77.3	74.8	80.3
	Utility	0.844	0.838	0.861	0.805	0.745	0.745	0.804
Male	VAS	80.8	85.3	79.5	78.8	78.3	74.6	79.6
	Utility	0.836	0.854	0.827	0.791	0.792	0.768	0.811
Total	VAS	81.8	82.6	82.1	80.7	77.8	74.7	79.9
	Utility	0.84	0.846	0.845	0.797	0.768	0.756	0.808

## Abbreviations

MO: Mobility
SC: Self-care
UA: Usual activities
PD: Pain/discomfort
AD: Anxiety/depression
VAS: Visual Analogue Scale
HRQoL: Health-Related Quality of Life
HUI: Health Utility Index
SF-6D: Short Form Six Dimensions
AQOL: Assessment of Quality of Life
SIP: Sickness Impact Profile
DCE: Discrete choice experiment
TTO: Time trade-off
CV: Coefficient of variation
IQR: Interquartile range

## References

[1] Patrick DL, Deyo RA (1989). Generic and disease-specific measures in assessing health status and quality of life. Med Care.

[2] Golicki D, Niewada M (2017). EQ-5D-5L Polish population norms. Arch Med Sci.

[3] Horsman J, Furlong W, Feeny D, Torrance G (2003). The Health Utilities Index (HUI®): concepts, measurement properties and applications. Health and Quality of Life Outcomes.

[4] Brazier J, Roberts J, Deverill M (2002). The estimation of a preference-based measure of health from the SF-Journal of. Health Econ.

[5] Sintonen H (2001). The 15D instrument of health-related quality of life: properties and applications. Ann Med.

[6] Hawthorne G, Richardson J, Osborne R (1999). The Assessment of Quality of Life (AQoL) instrument: a psychometric measure of Health-Related Quality of Life. Qual Life Res.

[7] Bergner M, Bobbitt RA, Kressel S, Pollard WE, Gilson BS, Morris JR (1976). The sickness impact profile: conceptual formulation and methodology for the development of a health status measure. Int J Health Serv.

[8] Rabin R, de Charro F (2001). EQ-5D: a measure of health status from the EuroQol Group. Ann Med.

[9] Puhan MA, Gaspoz J, Bridevaux P (2008). Comparing a disease-specific and a generic health-related quality of life instrument in subjects with asthma from the general population. Health Qual Life Outcomes.

[10] EuroQol Group. https://euroqol.org/eq-5d-instruments/.

[11] Marra CA, Woolcott JC, Kopec JA (1982). A comparison of generic, indirect utility measures (the HUI2, HUI3, SF-6D, and the EQ-5D) and disease-specific instruments (the RAQoL and the HAQ) in rheumatoid arthritis. Soc Sci Med.

[12] Sakthong P, Sonsa-Ardjit N, Sukarnjanaset P (2015). Psychometric properties of the EQ-5D-5L in Thai patients with chronic diseases. Qual Life Res.

[13] Kim TH, Jo MW, Lee SI, Kim SH, Chung SM (2013). Psychometric properties of the EQ-5D-5L in the general population of South Korea. Qual Life Res.

[14] Szende A, Janssen B, Cabases J (2014). Self-reported population health: an international perspective based on EQ-5D.

[15] Zrubka Z (2019). A comparison of European, Polish, Slovenian and British EQ-5D-3L value sets using a Hungarian sample of 18 chronic diseases. Eur J Health Econ.

[16] Herdman M, Gudex C, Lloyd A (2011). Development and preliminary testing of the new five-level version of EQ-5D (EQ-5D-5L). Qual Life Res.

[17] Prevolnik Rupel V, Srakar A, Rand K (2020). Valuation of EQ-5D-3L Health States in Slovenia: VAS based and TTO based value sets. Zdravstveno varstvo.

[18] Golicki D, Niewada M, Hout BV, Janssen MF, Pickard AS (2014). Interim EQ-5D-5L Value Set for Poland: First Crosswalk Value Set in Central and Eastern Europe. Value Health Reg Issues.

[19] R Core Team. R: a language and environment for statistical computing. R Foundation for Statistical Computing, Vienna, Austria (2019). https://www.R-project.org/.

[20] Yang Z, Busschbach J, Liu G, Luo N (2018). EQ-5D-5L norms for the urban Chinese population in China. Health and quality of life outcomes.

[21] Hinz A, Kohlmann T, Stöbel-Richter Y, Zenger M, Brähler E (2014). The quality of life questionnaire EQ-5D-5L: psychometric properties and normative values for the general German population. Qual Life Res.

[22] Purba FD, Hunfeld JA, Iskandarsyah A, Fitriana TS, Sadarjoen SS, Passchier J, Busschbach JJ (2018). Quality of life of the Indonesian general population: test–retest reliability and population norms of the EQ-5D-5L and WHOQOL-BREF. PLoS ONE.

[23] Scalone L, Cortesi PA, Ciampichini R, Cesana G, Mantovani LG (2015). Health related quality of life norm data of the Italian general population: results using the EQ-5D-3L and EQ-5D-5L instruments. Epidemiol Biostat Public Health.

[24] Poder TG, Carrier N, Kouakou CR (2020). Quebec Health-Related Quality-of-Life population norms using the EQ-5D-5L: decomposition by sociodemographic data and health problems. Value Health.

[25] Garcia-Gordillo MA, Adsuar JC, Olivares PR (2016). Normative values of EQ-5D-5L: in a Spanish representative population sample from Spanish Health Survey, 2011. Qual Life Res.

[26] Pattanaphesaj J, Thavorncharoensap M, Ramos-Goñi JM, Tongsiri S, Ingsrisawang L, Teerawattananon Y (2018). The EQ-5D-5L valuation study in Thailand. Expert Rev Pharmacoecon Outcomes Res.

[27] Bailey H, Janssen MF, La Foucade A, Kind P (2019). EQ-5D-5L population norms and health inequalities for Trinidad and Tobago. PloS One.

[28] Augustovski F, Rey-Ares L, Irazola V, Garay OU, Gianneo O, Fernández G, Ramos-Goñi JM (2016). An EQ-5D-5L value set based on Uruguayan population preferences. Qual Life Res.

[29] Nguyen LH, Tran BX, Le QNH, Tran TT, Latkin CA (2017). Quality of life profile of general Vietnamese population using EQ-5D-5L. Health Qual Life Outcomes.

[30] Prevolnik Rupel V, Erker Slabe R, Divjak M (2020). EQ-5D-3L population norms and health-related quality of life of orthopaedic patients in Slovenia. Value Health Region Issues.

